# A Rare Case of Simultaneous Brain and Leptomeningeal Metastases in a Patient With Pancreatic Cancer

**DOI:** 10.7759/cureus.79923

**Published:** 2025-03-02

**Authors:** Omar Al Zarkali, Mohammad Hussaini, Dae Won Kim, Hsiang-Hsuan Michael Yu, Paul Ott, Sarah Ali

**Affiliations:** 1 Internal and Hospital Medicine, Moffitt Cancer Center, Tampa, USA; 2 Hematopathology, Moffitt Cancer Center, Tampa, USA; 3 Gastrointestinal Oncology, Moffitt Cancer Center, Tampa, USA; 4 Radiation Oncology, Moffitt Cancer Center, Tampa, USA

**Keywords:** cerebral metastasis, leptomeningeal spread, pancreatic adenocarcinoma, pancreatic cancer, radiation therapy

## Abstract

Pancreatic cancer rarely metastasizes to the brain or meninges. Here, we describe a case in which a patient developed both. Our patient, a 75-year-old male individual, was diagnosed with pancreatic tumor on computed tomography (CT) of the abdomen and pelvis after presenting for abdominal pain. The patient underwent an endoscopic ultrasound-guided fine needle aspiration of the pancreatic mass and pathology was consistent with adenocarcinoma of the pancreas. A positron emission tomography (PET) scan showed metabolically active pancreatic head mass, retroperitoneal soft tissue nodule/lymph node, soft tissue thickening adjacent to the celiac artery, and within the anterior left lung hilum. He was treated with neoadjuvant chemotherapy followed by Whipple procedure due to presence of oligometastasis with great response to initial treatment. He later developed a frontal headache while on surveillance. MRI of the brain demonstrated multiple intraparenchymal metastases as well as leptomeningeal carcinomatosis. The patient underwent a posterior fossa craniotomy for resection of the mass. The pathology was consistent with metastatic pancreatic adenocarcinoma. This case describes a malignancy that presented with a unique form of metastasis.

## Introduction

Pancreatic cancer is the third leading cause of death related to cancers in men and women worldwide [1]. Mortality rates have increased by approximately 0.3% per year since 2000 in men and since 1975 in women. In 2024, over 66,000 patients were expected to be diagnosed with exocrine pancreatic cancer in the United States and over 50,000 patients were expected to die from the disease [2]. The majority of patients are diagnosed with advanced disease due to symptoms presenting after the cancer has spread [3]. Higher incidence of early detection has been noted largely due to surveillance guidelines of high-risk individuals. These include patients with certain genetic predispositions, family history, routine surveillance of incidentally detected pancreatic cysts, or early-stage cancer detected while undergoing abdominal imaging for unrelated reasons. Due to the majority of incidences being detected in late-stage disease, only a limited subset of patients is eligible for surgery. Prognosis remains poor despite surgery with 30% five-year survival of margin-negative pancreaticoduodenectomy and 10% for node-positive disease [4].

Patients with resectable cancer involving the pancreatic head or uncinate process (i.e., without evidence of local invasion or distant metastasis) undergo a pancreaticoduodenectomy, also known as a Whipple procedure [5]. Neoadjuvant chemotherapy can assist some patients with borderline resectable disease to achieve negative margins [6]. This can be done with chemotherapy or chemoradiotherapy. Neoadjuvant therapy can also be considered in patients expected to have longer recovery from surgery and thought to potentially have benefit from upfront chemotherapy to maximize total therapy received. Unfortunately, due to adverse effects from the neoadjuvant therapy, complications may exclude patients who were once eligible to receive surgery from having that option. Despite negative margins after having surgery, all patients are recommended to receive adjuvant chemotherapy therapy [7].

Pancreatic cancer metastasizes early in its disease course. Common sites are liver, lymph nodes, lung, peritoneum and bones [8]. It is more common to see pancreatic cancer metastatic to the skin as compared to either the brain or leptomeninges. One retrospective analysis from South Korea reviewed 1229 patients in their database and found only seven incidences of nervous system metastasis (0.57%) [9]. Four of these patients developed intraparenchymal brain metastases, three had spinal metastases, and none developed leptomeningeal diseases. Another retrospective study evaluated 5824 patients with metastatic pancreas ductal adenocarcinoma and found 25 patients who had developed brain metastasis (0.4%) [10]. Of these patients only four had developed leptomeningeal disease (0.07%). A retrospective analysis from Johns Hopkins reviewed 800 patients with pancreatic cancer and found only eight cases (1.0%) with brain metastasis [11]. Another similar retrospective study as well as a systemic review elucidated a handful of other cases of central nervous system (CNS) involvement of pancreatic cancer however without mention of leptomeningeal involvement [12-14]. Leptomeningeal metastasis is even less frequently seen than brain metastasis due to pancreatic cancer. Cases to date have only been documented in case reports and case series [15-31]. Cases with both brain metastasis and leptomeningeal disease are even rarer. Only four cases in our literature review discovered synchronous leptomeningeal disease with brain metastasis [16,21,26,30]. In this case report we discuss a patient with pancreatic cancer who developed metastases to both the brain and leptomeninges.

## Case presentation

**Figure 1:**
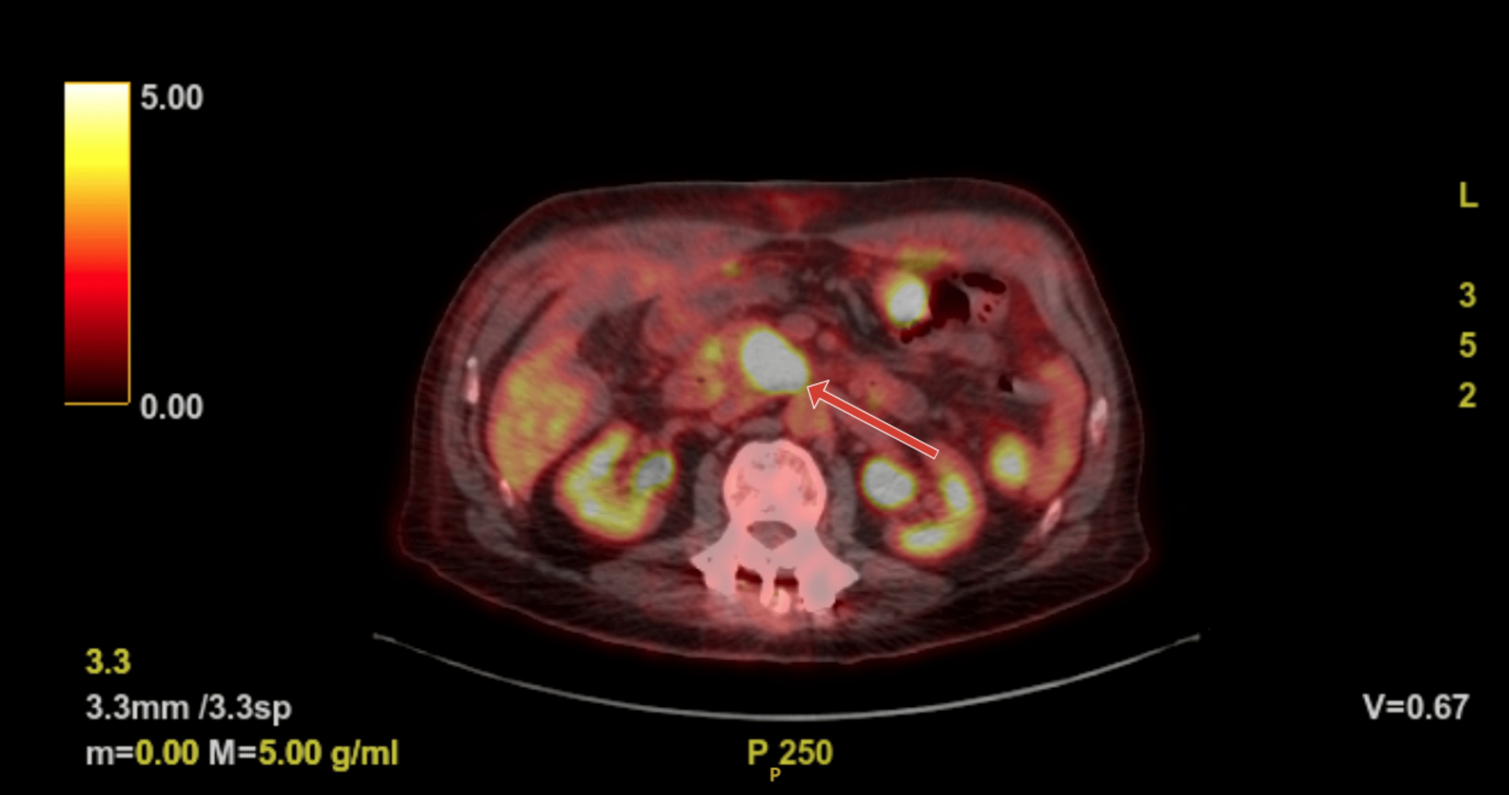
PET-CT, with red arrow pointing to hypermetabolic head of pancreas mass.

Our patient is a 75-year-old gentleman with a history of essential hypertension and benign prostatic hyperplasia who presented for evaluation of intractable abdominal pain. CT of the abdomen and pelvis was done to evaluate ongoing abdominal pain and it showed a prominent head of the pancreas. A follow-up MRI of the abdomen and pelvis demonstrated a lesion in the head and uncinate process of the pancreas measuring 2.6 cm with atrophy of the pancreas and dilatation of distal pancreatic duct concerning for primary neoplasm of the pancreas. The patient underwent an endoscopic ultrasound-guided fine needle aspiration of the pancreatic mass and pathology was consistent with adenocarcinoma of the pancreas. PET-CT re-demonstrated the pancreatic head mass and showed metabolically active retroperitoneal lymph node, soft tissue adjacent to celiac artery, and region within the anterior left lung hilum (Figure 1). There was also faint metabolic activity within a small right middle lobe nodule.

**Figure 2:**
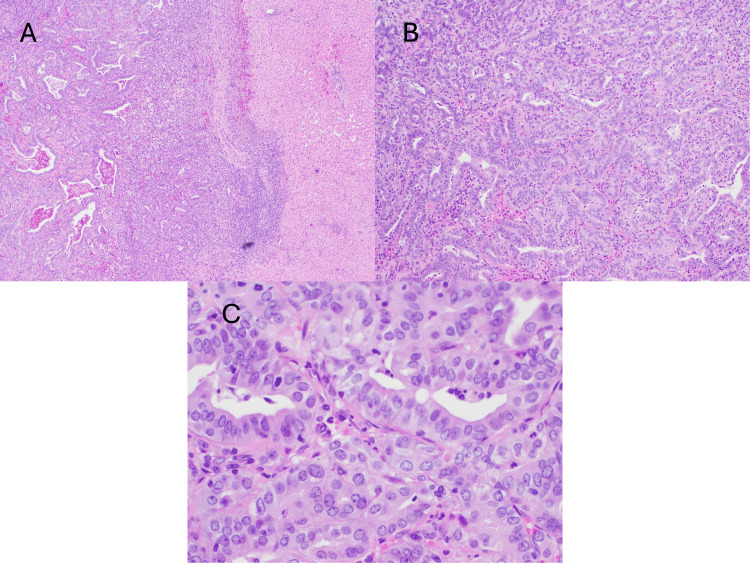
Liver biopsy; hematoxylin and eosin stain 4x (A), 10x (B), 20x (C), consistent with metastatic pancreatic adenocarcinoma.

He was taken by general surgery for exploratory laparotomy and Whipple procedure; however, he was found to have lesions on the liver that were not noted on initial imaging studies. A frozen section was sent to pathology intraoperatively and findings were consistent with metastatic adenocarcinoma (Figure 2).

**Figure 3:**
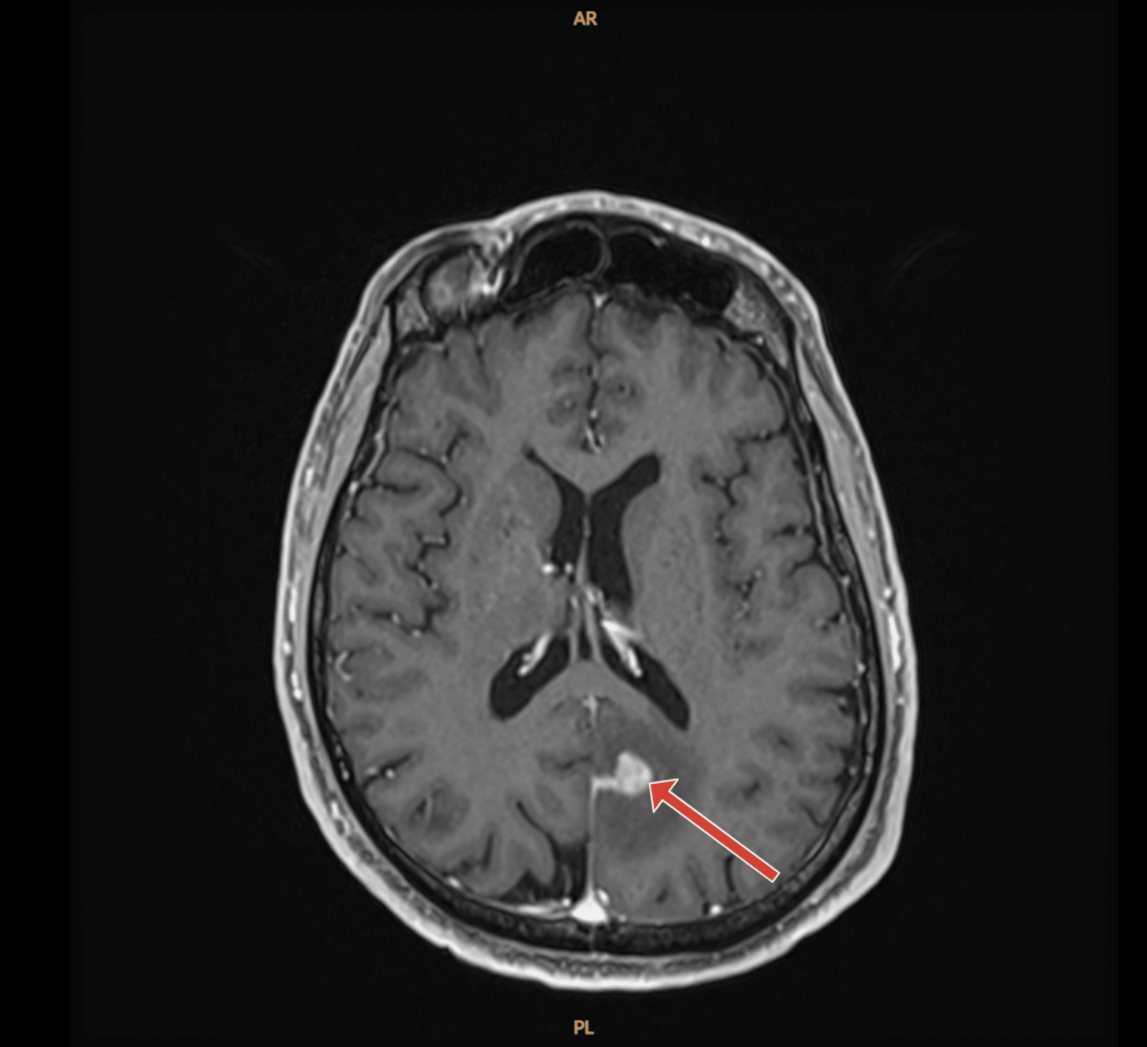
Post-contrast axial MRI T1-weighted of the brain, with red arrow pointing to marked mass like pial enhancement in the left parieto-occipital area concerning for leptomeningeal carcinomatosis.

**Figure 4:**
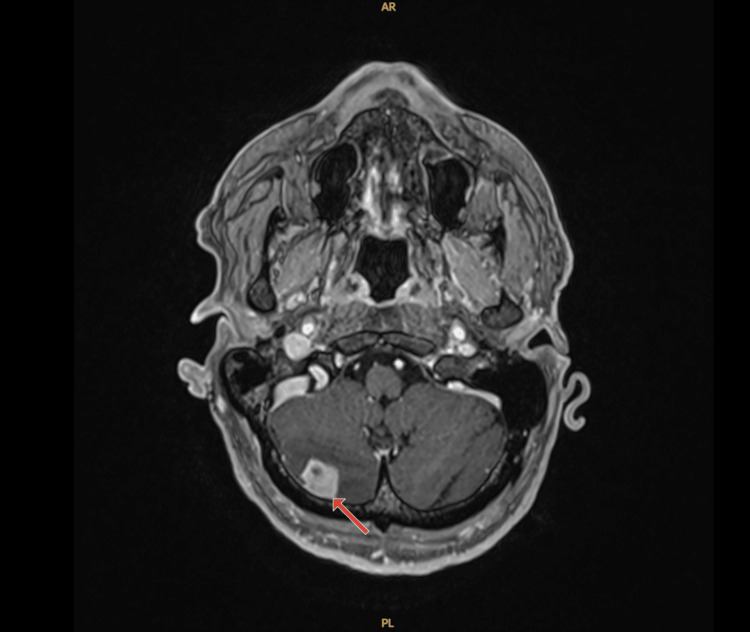
Post-contrast axial MRI T1-weighted of the brain, with red arrow pointing to lesion in the right cerebellar hemisphere consistent with intraparenchymal metastasis.

Due to the metastatic liver lesion, the Whipple’s procedure was aborted. Given his age, he was started on modified FOLFIRINOX (leucovorin calcium, fluorouracil, irinotecan hydrochloride, and oxaliplatin) and completed 12 cycles. Repeat CT thorax/abdomen/pelvis after completing chemotherapy showed no new lesions along with the decrease of already known lesions. There was difficulty delineating the pancreatic head mass which appeared indistinguishable from adjacent pancreatic tissue. Due to oligometastatic disease with exceptional response to the chemotherapy, he was re-evaluated for surgery and was found to be a suitable candidate for Whipple procedure. During his surgery he also underwent an omental pedicle flap and resection of the jejunal branch of the superior mesenteric vein due to adherence to the tumor. Final pathological staging post-operatively was T1aN0R0. He was deemed to have a complete response. After his surgery due to no evidence of disease, excellent response to neoadjuvant therapy, and his age, he was placed under surveillance without adjuvant chemotherapy. Within two months of starting surveillance, he developed a frontal headache. MRI of the brain was done and demonstrated left parietal 19 mm nodule, ring enhancing lesion in the right temporal lobe measuring 5 mm, an enhancement in the posterior right frontal lobe measuring 5 mm, and an enhancing nodule in the right cerebellum measuring 14 mm. After being evaluated by neurosurgery and discussed with radiation oncology, they decided to perform pre-operative radiosurgery followed by right posterior fossa stereotactic craniotomy with tractography for pathologic confirmation. The MRI at the time of radiosurgery treatment planning (10 days after the initial MRI), however, showed multiple intraparenchymal metastases in the right cerebellum, bilateral frontal lobes, and right occipital lobes as well as leptomeningeal carcinomatosis in the left occipital lobe, left parieto-occipital area, and posterior fossa (Figures 3, 4).

**Figure 5:**
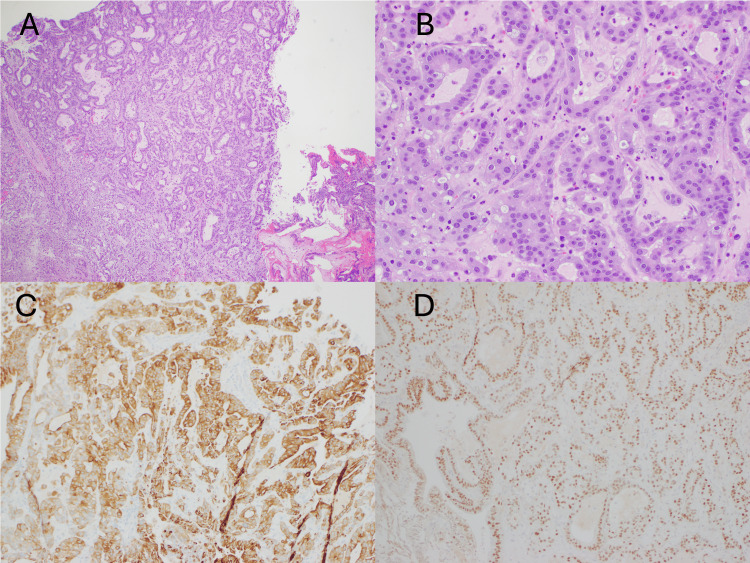
Right cerebellum biopsy; hematoxylin and eosin stain 4x (A), 20x (B), CK7 IHC (C), CDX2 IHC (D), consistent with pancreatic adenocarcinoma. IHC: immunohistochemistry.

Due to these findings, the recommendations changed to craniospinal irradiation (CSI) after craniotomy first for debulking and pathologic confirmation followed by intrathecal chemotherapy. The right cerebellar mass was resected. Pathology was consistent with adenocarcinoma of pancreatic origin (Figure 5).

Post-operative evaluation of the spine was done with imaging to assess for additional metastases. There was no evidence of leptomeningeal or spinal metastases on cervical, thoracic, or lumbar MRIs. He completed CSI successfully and an interval MRI demonstrated improvement in leptomeningeal disease without complete resolution. Prior to follow-up appointment with neurosurgery to discuss next steps regarding Ommaya implant for intrathecal chemotherapy, the patient had multiple admissions to an external hospital for multiple complications. His recovery from CSI was delayed due to hypotension and anemia requiring transfusions and two week-long hospital admissions. Two months after discharge he was readmitted due to encephalopathy and weakness. During his final admission he was found to have acute liver failure and hyponatremia due to SIADH (Syndrome of Inappropriate Antidiuretic Hormone Secretion). The patient had been recommended to transition to hospice during his final admission. Patient was lost to follow-up, however received notification of his death one week later. His most recent surveillance scan shortly prior to his multiple hospital admissions showed no evidence of extracranial metastasis.

## Discussion

Pancreatic cancer with metastasis to the brain or leptomeninges is an incredibly rare phenomenon with only a handful of documented cases. Developing both represents an even smaller subset of patients. Our in-depth literature review elucidated only 4 cases besides the one we present here. Due to the rarity of these cases, routine neuroimaging is not done on patients with pancreatic cancer, which can lead to cases being missed [32]. It is likely that due to the poor prognosis with even regional metastases of pancreatic cancer, patients do not live long enough to develop brain metastasis. With improved treatment options and earlier detection in many cases, it is possible to expect increased incidences of nervous system metastases in the future. It is worth noting however, that there were some cases found in our literature search in which patients did not have other sites of metastases besides the brain and leptomeninges at the time it was discovered, similar to our case [9-11]. This could reflect that neurological metastasis is simply an incredibly rare phenomenon and not a natural progression of the disease. On the other hand, an autopsy study revealed a brain metastasis rate of 7.9% in deceased pancreatic patients, which is a significantly higher rate than the ones in our literature review [33]. The majority of retrospective analyses found rates of brain and leptomeningeal metastasis of less than one percent. This further suggests that the rarity of these cases is likely due to their occurrences later in the disease course.

Our patient was diagnosed with brain metastasis and leptomeningeal disease 15 months after his first CT scan showed suspicious findings on his pancreas. Shortly after his first abdominal scan demonstrating the mass, he went for a Whipple’s procedure which confirmed stage IV disease. At the time the patient developed CNS and leptomeningeal disease, he had no evidence of extracranial disease. This is an incredibly rare and unusual phenomenon in a case with initial liver metastasis and exceptional response to chemotherapy. In 2015, the American Cancer Society reported an overall one- and five-year survival rate for pancreatic cancer of 28% and 7% respectively [34]. This represents all stages of disease. The five-year survival rates for regional and distant metastasis were 10% and 2% respectively. In 2024, they reported an overall five-year survival of 13% representing almost twice as many patients surviving for 5 years after diagnosis at any stage [2]. The five-year survival for regional and distant metastasis in the latter report is 16% and 3% respectively. While prognosis remains poor in pancreatic cancer, significant progress in outcomes has been made in the last decade, meaning once rarely seen complications in late disease may become more prevalent in the future. Our patient may have had microscopic CNS disease early in his disease state that was missed by conventional screening technology. A few decades ago, this patient could have passed prior to developing symptomatic neurological disease. Given his exceptional response to the chemotherapy and successful surgery, his survival increased long enough to allow this potentially microscopic disease to flourish and lead to his ultimate demise. This observation emphasizes the importance of learning to predict the course of the disease beyond what is typically observed. Special awareness is warranted in these patients who develop acute neurological symptoms that may be concerning for brain metastasis or leptomeningeal disease as early intervention with surgery, radiation, or intrathecal chemotherapy may be viable options if administered without delay.

## Conclusions

Metastatic pancreatic cancer remains a terminal illness despite advances in the recent history. As progress is made, it is important to learn to predict disease patterns beyond the typical course. Future advancements may lead to patients surviving with the disease longer than patients in the past. As outcomes continue to improve, new and previously unanticipated complications (including higher incidence of CNS disease) may warrant changes to screening guidelines and treatment options to further enhance outcomes in the field.
